# The Reasons and Associated Injuries Related to Baby Walkers Use Among Children in Riyadh, Saudi Arabia

**DOI:** 10.7759/cureus.17122

**Published:** 2021-08-12

**Authors:** Yasir A Albarrak, Abdulrahman F Algwaiz, Abdulelah M Sharaf, Albaraa A Alahmari, Alwaleed K Albadi, Ahmed M Almutairi

**Affiliations:** 1 College of Medicine, Imam Mohammad Ibn Saud Islamic University (IMSIU), Riyadh, SAU; 2 Pediatrics, Imam Mohammad Ibn Saud Islamic University (IMSIU), Riyadh, SAU

**Keywords:** baby walker, saudi arabia, injury, reasons, child safety

## Abstract

Background: Baby walkers (BW) are devices commonly used for helping babies’ mobility. However, it is associated with different types of injuries. Parents still use BWs and believe that it promotes early walking.

Objectives: This study aimed to estimate the use of baby walkers among children in Riyadh, Saudi Arabia, to identify the parental reasons for using BW, and to list their associated injuries.

Methods: This is a descriptive, cross-sectional study conducted between January 9, 2021, and January 31, 2021. A self-administered questionnaire was distributed through online means. We included all families living in Riyadh who have at least one child that is older than six months and younger than 36 months.

Results: This study included a total of 977 responders, of which, the majority 765 (78.3%) were baby walker users and 212 (21.6%) were non-users. Among those families using BW, the highest reason behind using them was to help the baby walk earlier (27.3%). However, believing that there is no need for using BW is the highest reason in the non-user group (29.40%). Fifteen percent of the responders reported that they had injuries related to BW usage and most of those injuries were falling downstairs (51.7%). The level of awareness regarding the disadvantage of BW was high in BW non-users (21.1%) compared to BW users (5.1%).

Conclusion: The results of our study show that the prevalence of BW usage is high in Riyadh, Saudi Arabia. The majority of the families lack knowledge of the benefits and hazards of BW. Thus, pediatricians and the Ministry of Health have to increase the awareness of BW. Furthermore, the government should ban their importation and selling.

## Introduction

Baby walkers (BW) are devices that have a seat supported by wheels that make babies who did not develop walking skills yet move around easily [1]. People have been using BW for their children since the early 1600s [2]. It is noticeable that the use of BW is popular among families from different communities around the world as this was apparent by many studies that were done in the last 30 years, which showed usage rates between 47% and 83% in infants younger than 15 months of age [3-7]. High prevalence rates of BW use correlate with high sale rates which were shown by a study that was done in the United States demonstrated that the sales of BW exceeded 600,000 pieces per year in 2005 with an estimated benefit of about 100 million dollars annually [8]. Parents gave many reasons behind the use of baby walkers, such as keeping the baby entertained, aid in the development of walking skills, allow time to rest from supervising the child, and "keeping the baby safe" [6,9-11]. On the other hand, the evidence that exists nowadays states that the use of BW slows down the development of independent walking and increases the chance that the baby will get injured [12,13]. The number of injuries reported to the National Electronic Injury Surveillance System (NEISS) of the US Consumer Product Safety Commission (CPSC) was 20,100 and 8800 in 1995 and 1999, respectively. Because of the mandatory and voluntary standards that were set by the CPSC for BW factories, the number of injuries declined significantly [14]. Moreover, NEISS reported 230,676 cases of children younger than 15 months who were treated for baby walker-related injuries in emergency departments in the period from 1990 to 2014 [15]. The American Academy of Pediatrics suggests a banning of production and sale of moveable BWs [1]. On April 7, 2004, Canada implemented the ban on import and sales of BW [16]. A comparison between BW users and non-users showed no significant variance in accelerating the acquisition of the skill of walking independently [17,18]. Another study found that BW could cause a delay in the development of walking skills [12]. The previous literature that is mentioned earlier showed the high prevalence of BW users that reflected the increasing numbers of injuries, we found that there is a lack of studies investigating this aspect in Saudi Arabia. Furthermore, we hope that this study will help the authorities in Saudi Arabia such as the Ministry of Health in knowing and estimating the number of BW-associated injuries in order to increase the campaigns and education of the public about the harms of its usage. In addition to that, this research could help the Ministry of Commerce to force the importers of BWs to set safety requirements and warning labels. In this study, we aimed first to estimate the prevalence of using BW among children in Riyadh, Saudi Arabia, second to identify the parental reasons for using BW, third to assess the awareness level about dangers and disadvantages of using BW, fourth to compare the parental attitude of using a BW with other general practices related to child safety, and fifth to list the BW associated injuries and their outcomes.

## Materials and methods

This is a descriptive, cross-sectional study approved by Institutional Review Board, King Saud Medical City (H1RE-26-Nov20-02). The study was carried out among the general population in Riyadh, Saudi Arabia, in the period from January 9, 2021, to January 31, 2021.

Riyadh is the capital city of Saudi Arabia and is considered the largest city among Arabic Gulf countries with a population estimate of over eight million. We targeted all families who are living in Riyadh. Our Inclusion criteria included all families who have at least one child who is older than six months which is the expected age to BW and younger than 36 months to get better recalling information regarding the usage of BW. We excluded any respondents who are not a first-degree relative to the child. Our final concluded sample size was 977 participants. Due to the circumstances related to the coronavirus disease 2019 (COVID-19) pandemic and the associated commitment to social distancing in order to contain the spread of the disease, we found it difficult and unsafe to interview the participants face to face. To overcome this problem, we took an action to distribute the questionnaire through online means. Consent was taken from the respondents when they agreed to fill the online survey. The self-administered questionnaire was developed in English and translated into Arabic. This questionnaire was designed and written by an expert pediatric consultant and the content was reviewed by other two pediatric consultants. To further assure the reliability and comprehension of the survey, a pilot study was performed with a sample size of 35 respondents. The questionnaire is divided into five parts: (i) demographic data of the parents and the targeted child (e.g., gender, age, level of education of the parents, and relation to the child); (ii) reasons and thoughts behind using and non-using BW; (iii) assess the level of BW awareness among the respondents by the following method - any respondent who answer the following two questions “baby walkers promote early walking” and “baby walker is safe for babies” correct was considered aware and respondent who answers one of the questions incorrectly was considered unaware; (iv) injuries associated with BW use; (v) assessment of general practices regarding the targeted child’s safety using eight questions - the questions were (1) have you ever used an infant car seat for this child (when he/she was an infant)? (2) Did you ever leave your child home without the supervision of an adult? (3) Did you or another adult ever share a bed with your child during sleeping? (When he/she was less than four months of age.) (4) Did you ever put any pillow in your child’s sleeping area? (When he/she was less than four months of age.) (5) Did you ever leave your child alone in the bathtub? (When he/she was an infant) (6) Are medications secured in a safe place (out of child’s reach) at home? (7) Is your child up to date on the recommended vaccines? (8) Does anyone smoke at the child’s home? - the targeted child’s safety score was as follows: optimal for those who answered eight questions correctly, good was seven to six, moderate was five to four, and poor was less than four. The gathered data were collected in a confidential manner to which only the research team will have access. Data were analyzed using SPSS version 23.0 (Chicago, IL: SPSS Inc.). The frequencies, percentages, mean, and standard deviation were conducted. Chi-square (χ^2^) was used to test the differences between the nominal data. The independent t-test was used to assess the differences between BW users and non-users. A p-value less than 0.05 was considered statistically significant.

## Results

A total of 977 people participated in the study including 765 BW users and 212 BW non-users, with the dominance of mothers’ participation nearly (≈60%) for both groups (p<0.05). For both groups, the majority of the mothers’ and fathers’ education levels were university/college with the BW users being higher (p<0.05). Further details regarding socio-demographic information are in Table 1. The participants were given a list of choices behind the specific reasons for using and not using BW. “To make the baby walk earlier” was the highest chosen reason behind using BW (27.3%), followed by “to be used for the baby's entertainment” (20.8%). “Received it as a present” was the least chosen reason behind using BW (3.7%). Pointed that the top reasons behind not using BW were “it is unnecessary” (29.40%), followed by “it is hazardous” (24.10%). “It makes the baby get bored easily” was the least chosen reason given behind not using BW (2.8%).

**Table 1:** Socio-demographic information (n=977) *P-value <0.05 BW: baby walkers; χ^2^/t/p: chi-square/independent t-test/p-value

Factor		BW user (765)	BW non-user (212)	χ^2^/t/p
What is your relationship with the child?	Father	125	43	5.738/0.13
		16.3%	20.3%	
	Mother	453	131	
		59.2%	61.8%	
	Brother	39	11	
		5.1%	5.2%	
	Sister	148	27	
		19.3%	12.7%	
Nationality	Saudi	709	174	21.467*/0.000
		92.7%	82.1%	
	Non-Saudi	56	38	
		7.3%	17.9%	
Residency	City	723	195	1.87/0.17
		94.5%	92.0%	
	Village	42	17	
		5.5%	8.0%	
Education/mother	Primary school	13	2	26.86*/0.000
		1.7%	0.9%	
	Intermediate school	23	1	
		3.0%	0.5%	
	Secondary school	128	30	
		16.7%	14.2%	
	University or college	460	107	
		60.1%	50.5%	
	Postgraduate	133	67	
		17.4%	31.6%	
	None of the above	8	5	
		1.0%	2.4%	
Education/father	Primary school	16	4	38.75*/0.000
		2.1%	1.9%	
	Intermediate school	27	4	
		3.5%	1.9%	
	Secondary school	127	11	
		16.6%	5.2%	
	University or college	395	97	
		51.6%	45.8%	
	Postgraduate	195	92	
		25.5%	43.4%	
	None of the above	5	4	
		0.7%	1.9%	
Mother's occupation	Working	439	143	6.99*/0.000
		57.4%	67.5%	
	Not working	326	69	
		42.6%	32.5%	
Child gender	Male	423	128	1.744/0.18
		55.3%	60.4%	
	Female	342	84	
		44.7%	39.6%	
First child?	Yes	228	122	55.57*/0.000
		29.8%	57.5%	
	No	537	90	
		70.2%	42.5%	
How many other children do you have	One child	71	23	9.20*/0.000
		13.2%	25.6%	
	Two children	154	22	
		28.7%	24.4%	
	Three children or more	312	45	
		58.1%	50.0%	
Age of participant (Mean±SD)		31.71±10.42	29.50±9.77	1.52/0.13
Age of child in months (Mean±SD)		19.18±17.24	19.08±17.28	0.08/0.94

**Figure 1:**
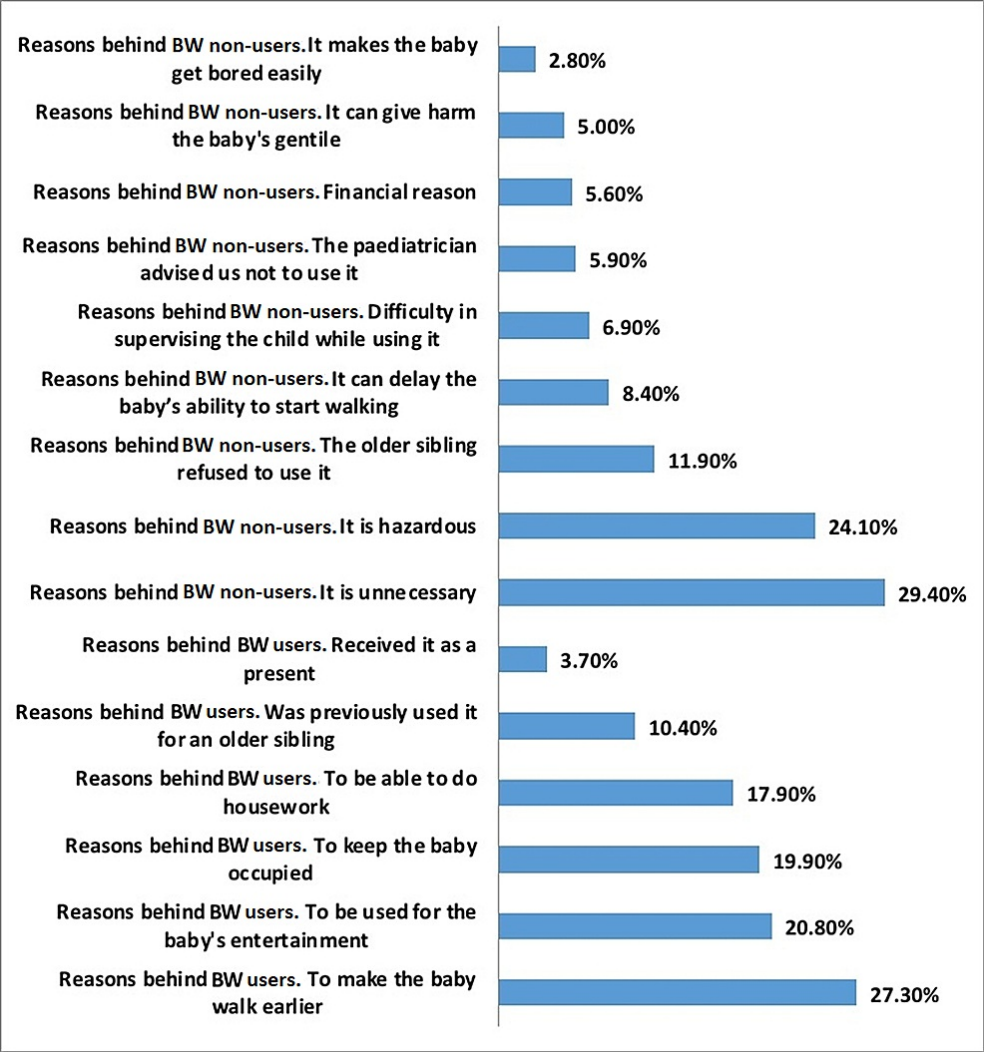
Percentages of reasons behind using and not using BW BW: baby walkers

Further details are in Table 2 and Figure 1. BW awareness was measured by two items (promotion of early walking and safeness), and the statements were classified into either “aware” or “unaware” participants for both groups. So, a chi-square test was conducted to test the level of awareness among BW users and non-users, the results indicate that there was a significant difference between BW users and non-users (χ^2^=54.95/p<0.05). Among all participants, a significant difference was found (χ^2^=669.89/p<0.05). The overall level of awareness was low, 8.6% of the participants were classified as aware, while the majority were classified as unaware (91.4%). A total of 21.1% of BW non-users were aware of the disadvantages of BW use as compared to 5.1% of BW users. 

**Table 2:** Reasons behind BW user and BW non-user BW: baby walkers

Reasons behind BW users	N	%	Reasons behind BW non-user	N	%
To make the baby walk earlier	513	27.3%	It is unnecessary	94	29.40%
To be used for the baby's entertainment	391	20.8%	It is hazardous	77	24.10%
To keep the baby occupied	373	19.9%	The older sibling refused to use it	38	11.90%
To be able to do housework	335	17.9%	It can delay the baby’s ability to start walking	27	8.40%
Was previously used it for an older sibling	195	10.4%	Difficulty in supervising the child while using it	22	6.90%
Received it as a present	69	3.7%	The pediatrician advised us not to use it	19	5.90%
			Financial reason	18	5.60%
			It can give harm the baby's gentile	16	5.00%
			It makes the baby get bored easily	9	2.80%
Total	1876	100%	Total	320	100%

Tables 3, 4 show caregiver’s attitude toward the child’s safety was measured by eight items. The items were answered “true” and “false,” so the possible score ranged between zero (the less relevant) and eight (the most relevant). The targeted child’s safety score was as follows: optimal for those who answered eight questions correctly, good was seven to six, moderate was five to four, and poor was less than four. The results confirmed that the caregiver’s attitude toward the child’s safety was moderate with a mean score (5.87±1.34), also there was an insignificant difference between users and non-user (t=1.73, p>0.05). Also, a chi-square test was conducted to assess the level of practice in each statement, it confirmed that the correct answers were the domains (p<0.000).

**Table 3:** Level of awareness towards BW among BW user and BW non-user *P-value <0.05 BW: baby walkers; χ^2^= Chi-square

No.	Statement	N/%	All			BW user		BW non-user		
			Aware	Unaware		Aware	Unaware	Aware	Unaware	
1	Baby walkers promote early walking	N	139	838		82	683	57	155	
		%	14.2%	85.8%		10.7%	89.3%	26.9%	73.1%	
2	Baby walker is safe for babies	N	308	669		208	557	100	112	
		%	31.5%	68.5%		27.2%	72.8%	47.2%	52.8%	
Total awareness		N	84	893		39	726	45	167	
		%	8.6%	91.4%		5.1%	94.9%	21.2%	78.8%	
		χ^2^=669.89*/0.000			χ^2^=54.95*/0.000					

**Table 4:** Caregiver’s attitude toward the child’s safety BW: baby walkers

Level	All		BW user		BW non-user	
	N	%	N	%	N	%
Optimal	101	10.3%	74	9.7%	27	12.7%
Good	538	55.1%	430	56.2%	108	50.9%
Moderate	275	28.1%	222	29.0%	53	25.0%
Poor	63	6.4%	39	5.1%	24	11.3%

**Figure 2:**
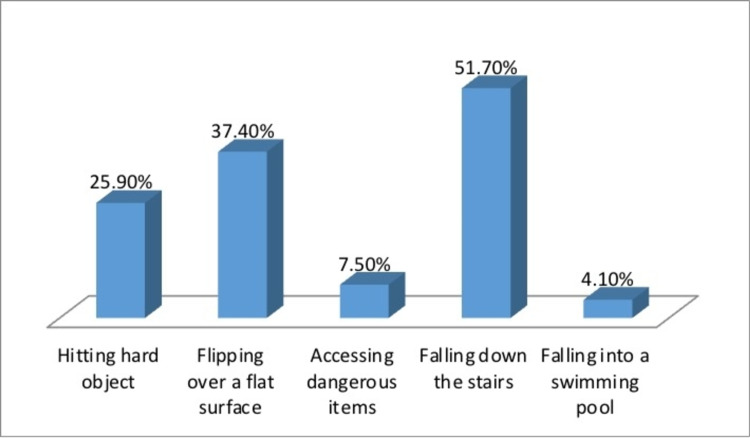
Mechanism of injury

**Figure 3:**
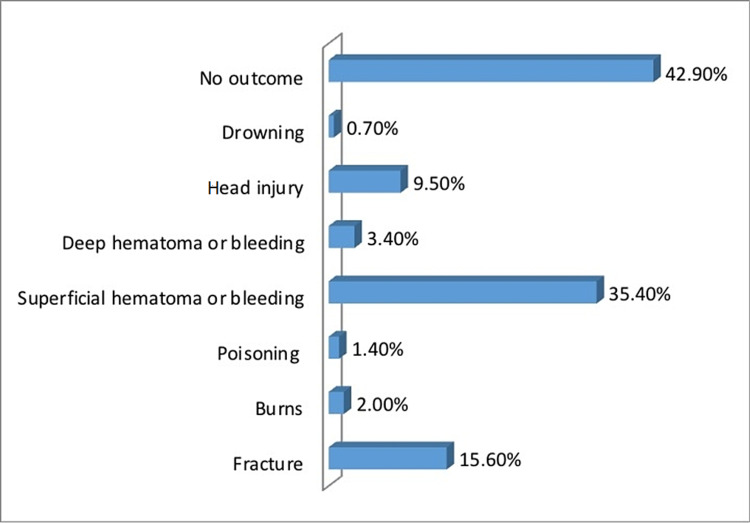
Outcomes of the injury

**Figure 4:**
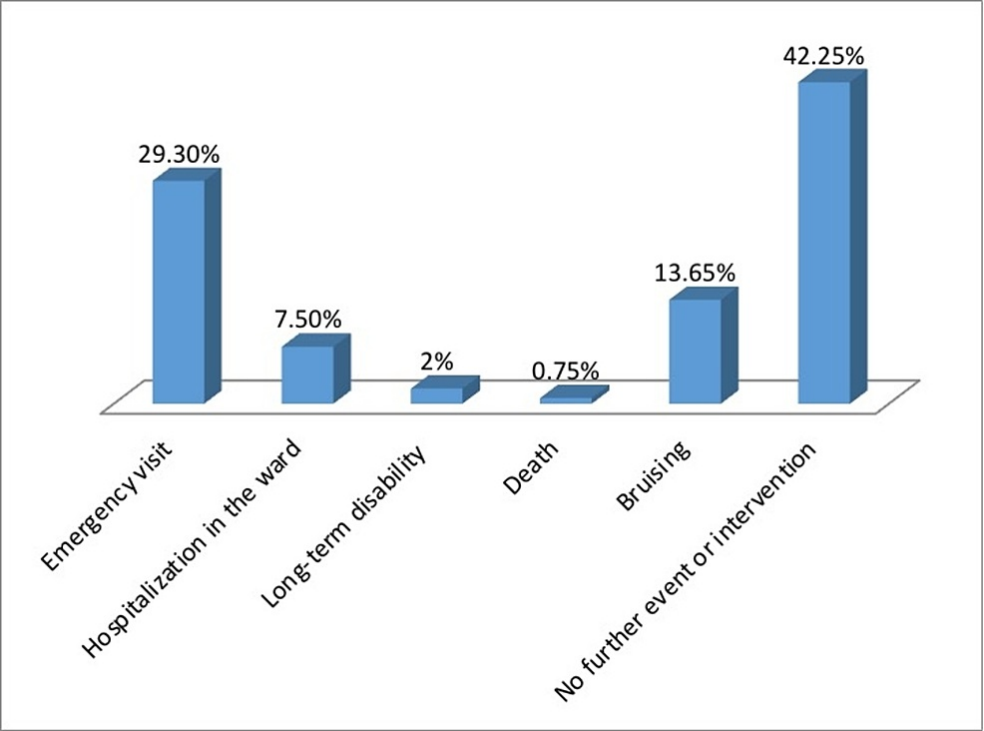
Further events or interventions

Table 5 shows that 15% of the children have been exposed to BW-related injury. This section included children who were exposed to an injury-causing event. As presented in Table 5 and Figure 2, the top two chosen mechanisms of injury were “falling down the stairs” (51.7%) and “flipping over a flat surface” (37.4%). “Falling into the swimming pool” was the least selected mechanism (4.1%). Table 5 and Figure 3 show the chosen outcomes of the children who were exposed to an injury. Most of them had no outcomes (42.9%). Superficial hematoma or bleeding (35.4%) was second. Drowning came last (0.7 %). Further events and interventions of the children who were injured are shown in Table 5 and Figure 4. The majority reported no further events and/or interventions were needed (42.2%). Emergency visit (29.3%) came second followed by bruising (13.6%), while death came last (0.7%).

**Table 5:** Baby walkers associated injuries and outcomes

Statement			N	%
Children exposed to an injury-causing event as a result of using a baby walker		No	830	85.0%
		Yes	147	15.0%
Falling into a swimming pool	Hitting hard object	No	109	74.1%
		Yes	38	25.9%
	Flipping over a flat surface	No	92	62.6%
		Yes	55	37.4%
	Accessing dangerous items	No	136	92.55
		Yes	11	7.5%
	Falling down the stairs	No	71	48.3%
		Yes	76	51.7%
	Falling into a swimming pool	No	141	95.9%
		Yes	6	4.1%
Outcome of the injury	Fracture	No	124	84.4%
		Yes	23	15.6%
	Burns	No	144	98.0%
		Yes	3	2.0%
	Poisoning (ingestion of chemicals, drugs, foreign body)	No	145	98.6%
		Yes	2	1.4%
	Superficial hematoma or bleeding	No	95	64.6%
		Yes	52	35.4%
	Deep hematoma or bleeding	No	142	96.6%
		Yes	5	3.4%
	Head injury	No	133	90.5%
		Yes	14	9.5%
	Drowning	No	146	99.3%
		Yes	1	0.7%
	No outcome	No	84	57.1%
		Yes	63	42.9%
Further events intervention	Emergency visit	No	104	70.7%
		Yes	43	29.3%
	Hospitalization in the ward	No	136	92.5%
		Yes	11	7.5%
	Long-term disability (describe further)	No	144	98.0%
		Yes	3	2.0%
	Death	No	146	99.3%
		Yes	1	0.7%
	Bruising	No	127	86.4%
		Yes	20	13.6%
	No further events and/or intervention	No	85	57.8%
		Yes	62	42.2%

**Figure 5:**
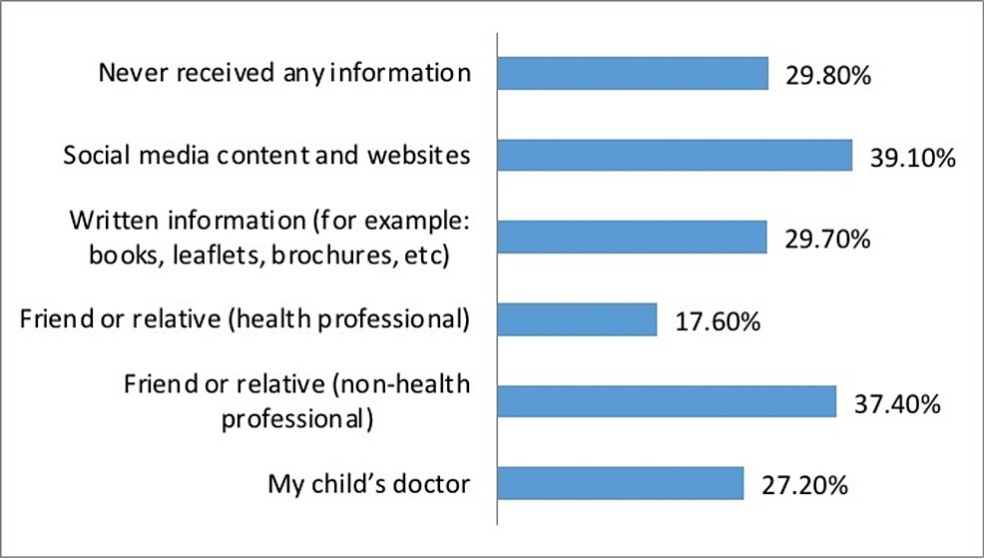
Source of caregivers' information

Table 6 and Figure 5 display the source of information regarding BW usage. A total of 39.1% had received their information from social media, followed by friends or relatives (non-health professional) (37.4%); 29.8% had never received any information.

**Table 6:** Source of caregivers' information

Source of information		N	%
My child’s doctor	Yes	208	27.2%
	No	557	72.8%
Friend or relative (non-health professional)	Yes	286	37.4%
	No	479	62.6%
Friend or relative (health professional)	Yes	135	17.6%
	No	630	82.4%
Written information (for example, books, leaflets, brochures, etc.)	Yes	227	29.7%
	No	538	70.3%
Social media content and websites	Yes	299	39.1%
	No	466	60.9%
Never received any information	Yes	228	29.8%
	No	537	70.2%

## Discussion

Despite the lack of evidence that supports the benefits of BW usage and the earlier literature which showed the harms of BW and its associated injuries, we noticed that the number of BW users is still increasing and companies keep marketing BW as a product that helps the baby to walk. Unfortunately, no specific medical association in Saudi Arabia has warned against BW use or run a public campaign to educate parents about the potential harms of using BW. In the present study, we aimed to identify the prevalence of BW users, possible reasons for using BW, and the associated injuries of its use. Moreover, the levels of awareness and safety practices among BW users and non-users were measured.

The prevalence of BW users in our study (78%) is similar to previous studies done in a high-income country like the United Arab Emirates and other middle-to-low regional countries that showed a prevalence rate between 54% and 87% [19-21]. Interestingly, most of BW non-users mothers (82.1%) have either a bachelor’s degree or postgraduate, while the percentage decreased in mothers of BW users to 77.5% with a significant p-value of <0.05. A similar result identified in a previous study that was done in Turkey stated that lower maternal education was considered a factor for using BW [20]. Although the difference in the level of education between BW users and non-users is found statistically, we found that both users and non-users gave a satisfying level of education with the non-BW users having a slightly higher level of education. With regards to this, we think that the level of education should not be taken as a strong factor regarding the decision of using BW in our study.

From the late 1990s till we established our study, parents gave the same common reasons behind using BW. For example, most of the parents in our study and previous studies believe that BW promotes early waking and keeps the baby entertained and occupied [6,19,20]. Although there is no evidence yet to prove their reasons, the evidence has been found to be against their beliefs. A previous study mentioned that a baby's development can be negatively affected by BW [12]. On the other hand, BW non-users in our study and previous studies reported the same reasons behind not using BW. For example, they think it is unnecessary and it could be hazardous. Pediatricians play a major role in everything related to a baby’s health; however, only 5% in our study and 7% in the previous study received advice from their pediatrician to not use BW [20].

Over the years, the issue behind the high numbers of BW users’ misconceptions is that the parents believe BW promotes early waking and is generally safe. These two misconceptions were noticed in our study by measuring the level of awareness. Most of the participants (91.4%) were unaware of the falsehood of these two misconceptions. Moreover, BW users were less aware compared to BW non-users. Similar results were noticed in an older study [4]. These two misconceptions are definitely wrong and the previous literature proved it [22].

In order to understand the general reason behind the huge prevalence of BW users, we investigated the other general safety practices towards the child. On one hand, the reason for this is to try to know if families who use BW have other unsafe general practices and it is expected for their use. On the other hand, to know the families that generally have safe practices and their decision to use BW was because of lack of knowledge about the harms of BW. Although there was no significant difference between BW users and non-users, we noticed more than half of BW users have good safety practices. This result can justify that the problem is related to the lack of knowledge and awareness regarding the use of BW. Conversely, an older study showed that BW users were more likely to have unsafe practices (e.g., leaving baby alone on high surface, using pillow in the baby’s bed, and having a hot drink while holding the baby) [23].

As there are high numbers of BW users in our study, it was expected to see associated injuries among the users. Furthermore, we also asked BW non-users about previous injuries in any of their other children. In this study, 147 children (15%) of both the BW user and older siblings of non-user group had been exposed to a previous injury in the past. Our results are considered high compared to 7.8% in Turkey and too low compared to 94% in Iraq [7,20]. The most common mechanism of injury in our study was falling down the stairs (51%), which may explain the need for a mandatory safety standard like fall protection breaks. Similar result was noticed by data extracted from National Electronic Injury Surveillance System (NEISS) [15]. Although most of the injured children did not have an outcome to their injury and did not require any further intervention, 35% of them had superficial bleeding and 29% were taken to the emergency department. Similar results were noticed among 646 injuries in a study in the United Arab Emirates, emergency visits were 118 of these [21].

As for other self-reported surveys, our study has several limitations. First, there is a potential for recalling bias regarding the use of infant walkers. We tried to minimize it by shorting the recall periods, asking only about the recent use of walkers, and including the current infant and toddlers. However, in the final part of our study, we included information about other siblings and old injuries which could be recalled incorrectly and misestimated. Another limitation could be related to the selection technique of the sample. The majority of the involved caregivers in our report are moderately to highly educated, influencing the final assessment. Different results might be seen if the survey is conducted among other less-educated populations. However, the level of knowledge among them would be expected to be less than our assessment.

We think educating and raising the level of awareness among Saudi caregivers is an essential step in controlling similar harmful practices. It could be achieved directly by involving the health care providers and pediatricians who need to discuss such injury prevention topics during the routine well-baby visits, specifically at the four to six-month visits. Moreover, the Ministry of Health and the Saudi Pediatric Association should have a role in this issue by stating an announcement and spreading awareness to the community about the harms and the disadvantages of BW. 

Higher steps need to be taken at the governmental level by forcing the importers of BW to put warning signs and make an effective system that protects the baby from associated injuries. And, if possible, holding the sales and importation of BW in Saudi Arabia by the Ministry of Commerce.

## Conclusions

There is an obvious widespread use of BW in our result. Moreover, participants gave different reasons behind using BW as it is safe, enjoyable, and promotes early walking. These misconceptions should be corrected by pediatrics medical associations primarily and babies’ physicians as they have a role in improving babies’ health. The Saudi authorities like the Ministry of Health and Ministry of Commerce should take a serious step to educate people about the potential harms of BW as well as to limit its sales.
